# Memoirs of a Southern Planter

**Published:** 1888-02

**Authors:** 


MEMOIRS OF A SOUTHERN PLANTER. By Mrs. Susan Dabney Smedes. Publishers: Cushings and Bailey, Baltimore.

The signal ability of the gifted author of this volume of 341 pages, in portraying the daily home life of the Southern Planter as it was prior to the great struggle which so changed the relations heretofore existing between master and servant in our Southern States, and which now appear as events of a past age, will be readily recognized by all conversant with the incidents of that period, on perusing this instructive and fascinating publication.

Its pages teem with scenes illustrative of southern life on the plantation, and describe the history of a true Southern gentleman, whose influence was recognized in the community of the Gulf States as that of a leader and counsellor. They also portray in simple yet impressive language, the strong ties which bound the better class of slaves to the families of their owners, and the care and anxiety manifested by the latter for the welfare of those thus entrusted to their protection by the peculiar nature of the institution which no longer exists in our land. This volume also gives a clear and correct insight into the workings of the institution of slavery in the United States concerning which so many writers have drawn distorted and unnatural pictures in attempts to produce sensational literature.

These memoirs by a Christian lady are both readable and instructive and cannot fail to greatly interest all who peruse them, and we commend them to those who desire to understand certain events of a period, which is rapidly becoming more and more obscure amid the busy and exciting scenes of this progressive age.

